# Synthetic Microbial Community Promotes Bacterial Communities Leading to Soil Multifunctionality in Desertified Land

**DOI:** 10.3390/microorganisms12061117

**Published:** 2024-05-30

**Authors:** Xinwei Hao, Yazhou Gu, Hongzhi Zhang, Xiao Wang, Xiaozhen Liu, Chunlei Chen, Congcong Wang, Xiaoqing Zhang, Xingyu Liu, Xihui Shen

**Affiliations:** 1 State Key Laboratory for Crop Stress Resistance and High-Efficiency Production, Shaanxi Key Laboratory of Agricultural and Environmental Microbiology, College of Life Sciences, Northwest A&F University, Xianyang 712100, China; xinweihao1995@163.com (X.H.); wangxiaoyx@nwafu.edu.cn (X.W.); 15612250872@163.com (C.C.); wangcc@nwafu.edu.cn (C.W.); 2 Qingyang Longfeng Sponge City Construction Management and Operation Co., Ltd., Qingyang 745000, China; gyz0916@sina.com (Y.G.); 15688943689@163.com (H.Z.); 3 Institute of Grassland Research, Chinese Academy of Agricultural Sciences, Hohhot 010013, China; xiaozhenliu88@163.com (X.L.); zhangxiaoqing@caas.cn (X.Z.); 4 State Key Laboratory of Geological Processes and Mineral Resources, Institute of Earth Sciences, China University of Geosciences, Beijing 100083, China; wellwoodliu@163.com

**Keywords:** desertified land, synthetic microbial community, soil microbiome, nutrient cycling, soil multifunctionality

## Abstract

Soil desertification is an important challenge in global soil management, and effectively and stably restoring soil function is an urgent problem. Using synthetic microbial communities (SynComs) is a burgeoning microbial strategy aimed at enhancing soil nutrients through functional synergies among diverse microorganisms; nevertheless, their effectiveness in restoring desertified soils remains unknown. In this study, we conducted a two-year field experiment using a SynCom constructed by in situ probiotic bacteria and set up control, chemical fertilizer, and combined SynCom–chemical fertilizer (combined fertilizer) treatments to investigate the linkage between microbial communities and soil multifunctionality in the soil surface layer (0–10 cm). Both the bacterial and fungal communities differed the most under the combined fertilizer treatment compared to the control. The bacterial communities differed more under treatments of the SynCom than the chemical fertilizer, while the fungal communities differed more under the chemical fertilizer treatment than the SynCom treatment. Regarding soil function, the SynCom strengthened the correlation between enzyme activities and both bacterial communities and functional properties. pH and available potassium were the main influencing factors under the chemical fertilizer and combined fertilizer treatments. The beta-diversity of the bacterial communities was significantly correlated with soil multifunctionality. Random forest analyses showed that the SynCom significantly enhanced the bacterial communities, driving soil multifunctionality, and that some potential microbial taxa drove multiple nutrient cycles simultaneously. In summary, the SynCom effectively increased the abundance of most carbon, nitrogen, and phosphorus functional genes as well as soil enzyme activities. The bacterial community composition contributed significantly to soil multifunctionality. Hence, the development of novel microbial agents holds significant potential for improving soil functionality and managing desertification.

## 1. Introduction

Land desertification, characterized by a decrease in soil cover and a gradual loss of ecological protection, is particularly prevalent in dry, semi-arid, and subhumid locations.

External disturbances can easily disrupt fragile ecosystems, leading to soil nutrient imbalances and reduced fertility, thereby restricting plant growth. Fractional vegetation cover is crucial for controlling desertification. Measures such as setting up sand barriers, converting farmland to forests, and implementing grazing bans have achieved significant results in Northwest China [1]. While these natural restoration measures can slow desertification and restore vegetation growth, they pose challenges by being time-consuming and reducing food production [2,3]. Meanwhile, recent studies have confirmed that artificial vegetation restoration is more conducive to ecologically sustainable development [4]. According to local climate conditions and geographical environments, proper land management can optimize soil properties and increase vegetation productivity. Furthermore, artificial vegetation restoration has the advantages of a short cycle and strong targeting. Therefore, it is imperative to identify favorable measures to promote vegetation recovery for the ecological restoration of desertification.

Chemical fertilizer is a frequently employed method in modern agriculture to provide nutrients essential for plant growth by increasing mineral nutrients. For example, in nitrogen-deficient desertified grasslands, the targeted application of nitrogen fertilizer can restore the growth of dominant grass species [5]. Yet, the rational utilization of chemical fertilizers is a challenge. Prolonged use or improper nutrient ratios can easily pollute the environment and accelerate soil degradation [6]. The development of microbial agents offers novel solutions for agricultural practices. Soil microorganisms can promote nutrient cycling and improve land productivity. For example, plant-growth-promoting rhizobacteria (PGPR) enhance nutrient acquisition, regulate physiological metabolism, and improve plant adaptability. Recently, synthetic microbial communities (SynComs) have emerged as a promising strategy, utilizing indigenous beneficial strains to enhance plant growth through functional integration [7,8]. Unlike commercial microbial inoculants, the microbial members of SynComs are better able to colonize the initial environment and interact with plants to achieve a “home-field advantage” [9]. An increasing body of research evidence indicates SynComs’ usefulness in enhancing plant productivity and resilience, especially in low-fertility areas [10,11]. SynComs can lead to a reduction in chemical fertilizer inputs and promote environmental sustainability. However, little is known about the effectiveness of SynComs in desertification management, and few research studies have looked specifically at their effects on soil biological functions.

The functional characteristics and ecological niches of different microbial types sustain a wide range of ecological functions. According to studies, soil moisture has a substantial impact on the composition and function of soil microbial communities. Especially in desert soils, moisture is a key factor limiting microbial growth and metabolic activities. Increasing soil moisture not only increases microbial biomass, but also promotes organic matter decomposition, thereby improving soil fertility [12,13]. Different fertilization practices affect soil multifunctionality by altering microbial communities. Several studies have shown that the application of mineral fertilizers enhances microbial biomass and diversity and is more likely to affect fungal community structure [14]. However, irrational fertilization can inhibit microbial activity and reduce microbial communities’ ecological functions. SynComs restructure healthy microbial communities by introducing core functional strains [15]. A rising number of studies have proven that the application of SynComs enhances inter-root microbial synergism and improves plant productivity [16]. Positive correlations between microbial diversity and ecological function have been demonstrated in several ecosystems [17], with species richness favoring ecological function recovery. Microbial β-diversity, an important feature of community composition, has received little attention in studies involving ecological multifunctionality [18]. Therefore, whether the changes in microbial communities generated by SynComs are conducive to improving soil multifunctionality is key to their application. This knowledge is necessary for microbial ecological remediation and the restoration of ecosystem functionality in the context of global land degradation.

As reported herein, we conducted a field experiment in a desertified area of Northwest China to investigate the association between microbial populations and soil multifunctionality at the soil surface (0–10 cm) using chemical fertilizer, SynCom, and SynCom–chemical fertilizer treatments. We hypothesized that the SynCom would alter the microbial communities to enhance soil multifunctionality, and that the bacterial communities would be more affected than the fungal communities. The objectives of this study were (1) to assess the ecological effects of SynComs in desertification management, and (2) to elucidate the link between microbial communities and ecological functions under different treatments. Our results demonstrated the critical role of microbial communities in desertification restoration and the positive effects of SynComs on ecological multifunctionality. This provides new ideas for solving environmental problems and contributes to ecological sustainability.

## 2. Materials and Methods

### 2.1. Field Experiments and Soil Sample Collection

**Figure 1:**
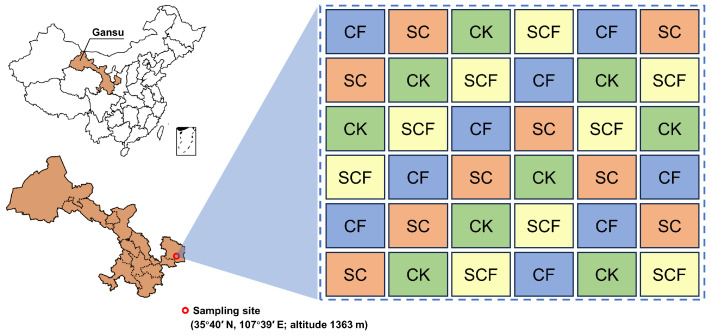
Field experiment location and sample plot layout. There were four different treatments, each with 9 replicate plots randomly distributed, for a total of 36 plots.

Field experiments were conducted over two years (May 2019 to June 2021) in a desertified area on the Loess Plateau (35°40′ N, 107°39′ E) in Qingyang City, Gansu Province, China. The local soil is classified as Calcaric Cambisols [19]. The area’s altitude is 1363 m, with a mean annual temperature of 10 °C and annual rainfall of 450 mm. The area is a typical desertified area with sparse vegetation (less than 30% coverage), fragile ecological environment, severe soil erosion and wind erosion, and low natural restoration capacity. The particle size composition of the soil was as follows: 60% sand (2.0–0.05 mm), 25% chalk (0.05–0.002 mm), and 15% clay (<0.002 mm). The four treatment groups were as follows: (1) unfertilized control (CK); (2) chemical fertilizer (CF); (3) SynCom (SC); (4) combined SynCom–chemical fertilizer (SCF). Each treatment had nine replicate plots (50 m² each), totaling 36 randomly arranged plots (Figure 1). The control group was the unfertilized treatment replacing the added substance with an equal volume of water. Chemical fertilizer is water-soluble compound fertilizer (nitrogen–phosphorus–potassium = 4:3:3, total nutrient ≥ 45%), with an application rate of 150 kg/ha/year. The strain members of the SynCom were isolated from the inter-root soils of the plants in this desertification area region, and the combination was adjusted based on functional features and compatibility (Figure S1 and Table S1). Specifically, SynCom consisted of five bacterial strains with specific functions belonging to the genera *Arthrobacter*, *Bacillus*, *Acinetobacter*, *Pseudomonas*, and *Mixta*. These bacterial isolates have more than two life-promoting functions. In addition, they are very compatible in culture and can be combined to synergize their functional roles. The candidate strains were stored in −80 °C refrigerator. Before the experiments, an activated single colony of each strain was picked and incubated with tryptic soy broth (TSB) medium overnight. The concentration was adjusted to 2 × 10^8^ CFU/mL after washing twice with sterile water and finally mixed in equal proportions to make bacterial suspension. The final concentration of the SynCom was 1 × 10^9^ CFU/mL. The combined fertilizer was 50% chemical fertilizer and 50% SynCom, mixed well and sprayed in the same volume. The field experiments were conducted in accordance with the conventional local management methods.

We collected soil samples two years after the field experiment. Soil samples were collected using a 5 cm diameter soil auger. Soil from five randomly selected points in each replicate plot was mixed to form a composite sample. In addition, we collected the top layer of soil at three different depths of 0–2 cm, 2–5 cm, and 5–10 cm for the study. A total of 108 soil samples (4 treatments × 3 soil depths × 9 replicate plots) were collected for this study. Soil samples were transported to the laboratory on dry ice, with one portion stored at −80 °C for DNA extraction and the other at 4 °C for determining soil enzyme activity and physicochemical parameters.

### 2.2. Soil Physicochemical Properties and Enzyme Activities

Soil physicochemical properties were determined by air drying the soil for two weeks. Standard assay methods [20] were used to test all soil physicochemical properties. Soil pH was measured using a pH meter (Mettler Toledo, Columbus, OH, USA). Soil electrical conductivity (EC) was measured using a conductivity meter (Raymarine, Fareham, UK). Soil organic matter (SOM) was measured using a total organic carbon analyzer (Shimadzu, Kyoto, Japan). Soil total nitrogen (TN), total phosphorus (TP), total potassium (TK), and available nitrogen (AN) were measured using an automatic flow analyzer (Seal). Available phosphorus (AP) and available potassium (AK) were determined using flame photometer (Sherwood, Cambridge, UK).

Soil enzyme activities were determined using Elsas’s method [21]. β-glucosidase activity (BG) was expressed by assaying *p*-nitrophenol (PNP) released per hour per gram of soil. Dehydrogenase activity (DHA) was expressed by assaying 2,3,5-triphenyl formazan (TPF) released per hour per gram of soil. Alkaline phosphatase activity (ALP) was expressed by assaying the PNP released per hour per gram of soil under alkaline conditions (pH = 11). Urease activity (UA) was expressed by determining the amount of ammonia released. All enzyme activities were finally determined using microplate labeler (BioTek, Winooski, VT, USA) with the corresponding absorbance values.

### 2.3. Soil DNA Extraction and Amplicon Sequence Analysis

Soil genomic DNA was extracted from 0.5 g of fresh soil using the FastDNA™ SPIN Kit (MP Biochemicals, Santa Ana, CA, USA) following the manufacturer’s instructions. Bacterial communities and fungal communities were analyzed by amplicon sequencing. Each sample was individually amplified three times to reduce errors. For the bacterial community [22], primers 515F and 907R were used to amplify the V4–V5 region of the 16S rRNA gene. For the fungal community [23], primers ITS3-F and ITS4-R were used to amplify the ITS2 region. The primers used all contain sample-specific barcode. Finally, high-throughput sequencing of purified amplicons was performed using the MiSeq PE300 platform of Majorbio Bio-Pharm Technology Co., Ltd. (Shanghai, China).

The obtained raw sequences were analyzed using the EasyAmplicon pipeline [24]. The raw sequences were merged to excise the double-ended primers and barcode, followed by quality control (quality ≥ 20, VSEACH 2.7.1), denoising (miniuniqusize > 10, UNOISE3), and chimera removal (SILVA 123) to obtain the original amplicon sequence variant (ASV) table. Based on the Bayesian algorithm, the taxonomic information of bacterial and fungal ASVs was obtained using the SILVA (v.123) database and the UNTE (v.8.2) database, respectively. We removed ASVs that did not include bacterial and fungal information. The sequencing data of each sample were sufficient for subsequent analyses (Figure S3). The bacterial and fungal sequences of each sample were diluted to the same number (bacterial depth: 4708; fungal depth: 1675) by random sampling. The final ASV table we obtained was used for subsequent microbial analyses.

### 2.4. Quantitative Real-Time PCR (qRT-PCR)

Quantitative PCR for soil functional genes was performed using a LightCycler^®^ 96 instrument (Roche, Indianapolis, IN, USA). Nitrogen cycle functional genes are involved in nitrogen fixation (*nifH*), organic nitrogen mineralization (*chiA*), ammonia oxidation (*AOA* and *AOB*) and denitrification (*nirK*, *nirS*, *nosZ,* and *qnorB*), respectively. Carbon cycle functional genes are involved in carbon fixation (*cbbM*) and carbon degradation (*Fungcbhif* and *GH31*). Phosphorus cycle functional genes were involved in organic phosphorus mineralization (*phoD*). The primer information and reaction procedure of these functional genes (Table S4) were based on previous research [25,26]. Standard curves (R^2^ > 0.99) for each gene were plotted using a gradient dilution of the plasmid with the target gene. Three replicates were performed for each gene in each test sample to reduce error. Finally, the number of functional genes in each sample was estimated using the number of threshold cycles and the standard curve.

### 2.5. Assessing Soil Multifunctionality

Soil multifunctionality involves the simultaneous quantification of multiple ecosystem services, including nutrient supply, organic matter degradation, and elemental cycling [27]. In this study, we quantified 20 soil functionality indicators. Among them, five indicators were related to the carbon cycle (SOM, BG, DHA, carbon fixation genes, and carbon degradation genes); seven indicators were related to the nitrogen cycle (TN, AN, UA, nitrogen fixation genes, nitrogen mineralization genes, nitrification genes, and denitrification genes); and four indicators were related to the phosphorus cycle (TP, AP, ALP, and phosphorus mineralization genes). The remaining four indicators were TK, AK, pH, and EC. Referring to Zhang’s method [28], we standardized all the individual functional indicators (ranging from 0 to 1) and then averaged them as indicators of soil multifunctionality. In addition to this, we also calculated the carbon, nitrogen, and phosphorus cycle multifunctional indicators based on the classification of functional indicators.

### 2.6. Data Analysis

Data analyses were performed using R software (version 4.1.1). The data were subjected to normal distribution, and one-way analysis of variance (ANOVA) was used to test the differences in microbial alpha-diversity, functional gene abundance, physicochemical properties, and enzyme activities across treatments. Significance of differences was determined using the least significant difference (LSD) test and Tukey’s post hoc test. Microbial alpha-diversity (Shannon index) and beta-diversity were calculated using the “-alpha_div” and “-beta_div” commands within Usearch software (version 11.0.1), respectively. The significance of community structure between different subgroups was also tested by analysis of similarities (ANOSIM). Stress value was used to assess the validity of the NMDS model, which is usually less than 0.2 to prove validity. Grouped alluvial diagrams for the top 10 phyla of bacteria and fungi were drawn using the R package ‘galluvial’ (version 0.12.5). Clustered heatmaps for the top 40 genera of bacteria and fungi were realized with the R packages ‘pheatmap’ (version 1.0.12) and ‘RColorBrewer’ (version 1.1-3). The heatmap clustering scheme was the Bray–Curtis distance metric and the complete clustering method. The volcano maps were drawn with the R packages ‘ggplot2’ (version 3.5.1) and ‘ggrepel’ (version 0.9.1), with colors indicating different phyla and traits indicating enrichment types. Stacked bar graphs were generated with the R package ‘ggplot2’ (version 3.5.1) to represent the distribution of enriched ASVs at the phylum level. Mantel analysis visualization was achieved using the R package ‘linkET’ (version 0.0.7.4). In this, heatmaps presented the Pearson correlation and significance between environmental factors.

Linear regression was used on the scatter plot to demonstrate the association between microbial community and soil multifunctionality, and the significance test was carried out using the R package ‘ggpubr’ (version 0.6.0). Random forest (RF) model was calculated by R package ‘rfPermute’ (version 2.5.2) to assess the importance of microbial community and microbial taxa on soil multifunctionality. The percentage of mean squared error (MSE) represented the importance scores of the different variables, while the significance of the variables was assessed by 1000 random permutations. Finally, full model significance was assessed using ‘A3’ package (version 1.0.0) based on 1000 random permutations. R^2^ denotes the variance explained by the full model, in which a higher value represents a better model fit.

## 3. Results

### 3.1. Variation in Bacterial and Fungal Community Diversity under Different Treatments

**Figure 2:**
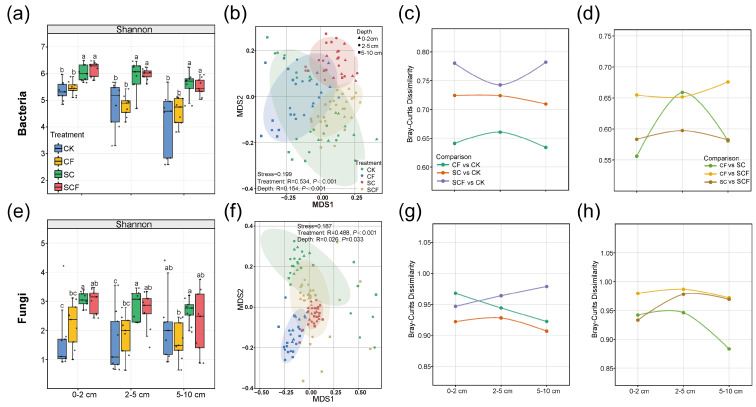
Shannon index of bacteria (**a**) and fungi (**e**) for various treatments and soil layers. Different letters indicate sig-nificant differences (*p* < 0.05) among treatments. Beta diversity of bacterial (**b**) and fungal (**f**) communities (based on Bray–Curtis distances) varied with treatment and soil layer. Stress values of less than 0.2 for non-metric multidimensional scaling (NMDS) plots indicate reliable results. The analysis of similarities (ANOSIM) tested the significance of the effects of treatments and soil layers on the microbial communities separately. Community dissimilarity revealed the effect of soil layer variation on microbial communities. Community dissimilarity in fertilizer–control comparisons (**c**,**g**) and fertilizer–fertilizer comparisons (**d**,**h**) in bacterial and fungal communities.

According to the amplicon sequencing results of all of the samples (Figure S2), we obtained 4708 bacterial ASVs and 1675 fungal ASVs, respectively. The SC treatment significantly increased the alpha-diversity of the bacteria and fungi at different soil depths compared to the CK treatment (Figure 2). Meanwhile, the SCF treatment also enhanced microbial diversity, but its effect was weaker than that of the SC treatment. The CF treatment showed a relatively consistent level of bacterial alpha-diversity and an increased level of fungal diversity compared to those of the CK treatment (Figure 2a,e). The effect of the fertilizer treatments on the bacterial and fungal communities was more pronounced compared to the soil layers (Figure 2b,f). To reveal changes in microbial community differences with soil depth, we calculated the Bray–Curtis dissimilarities between the treatment groups. The SCF treatment exhibited the greatest microbial community variability compared to that of the control (Figure 2c,g). Notably, the SC treatment induced a greater level of variability in the bacterial communities, while the CF treatment had a more pronounced effect on the fungal communities. In addition, the microbial community differences between the SC and CF treatments reached a maximum at 2–5 cm (Figure 2d,h). The community differences between the SCF and SC treatments were even smaller in a comparison between the treatment groups. 

### 3.2. Differences in Microbial Composition under Different Treatments

**Figure 3:**
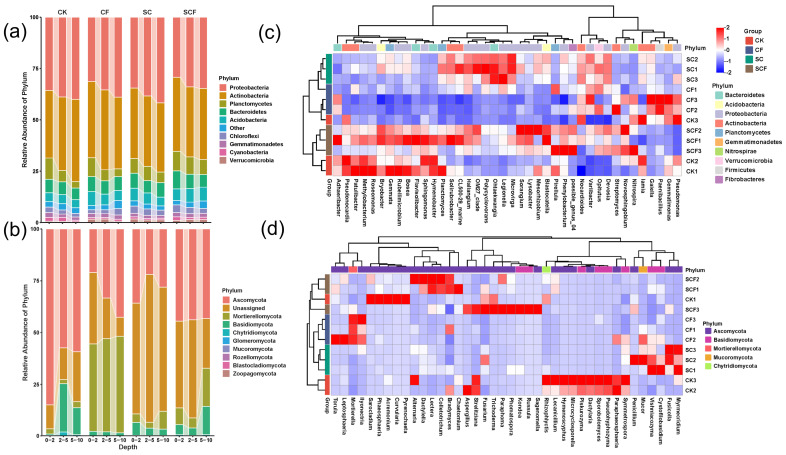
Bacterial and fungal community composition among treatments. Grouped stacked plots showed changes in relative abundance of the top 10 phyla of bacteria (**a**) and fungi (**b**). Clustering heatmaps demonstrated bacterial (**c**) and fungal (**d**) community composition differences at the genus level (top 40). Horizontal clustering demonstrated similarities between samples, and vertical clustering demonstrated phylogenetic relationships between microbial genera.

Proteobacteria and Actinobacteria were the dominant bacterial phyla, accounting for 68.6–80.1% of all reads (Table S2), and their relative abundance increased with the soil depth (Figure 3). For fungi, the dominant phylum differed under different treatments (Table S3). Ascomycota was the dominant phylum in the CK treatment (an average of 67.2%), and Mortierellomycota was the dominant phylum in the CF treatment (an average of 44.7%); however, an unassigned phylum dominated in the SC treatment (an average of 61.6%) (Figure 3a,b). Differential bacteria mainly belonged to Proteobacteria and Actinobacteria, and differential fungi mainly belonged to Ascomycota and an unassigned phylum (Figures S3 and S4). The SC treatment produced the highest number of differential microorganisms. The SCF treatment enriched differential microorganisms the most (in terms of percentage). The results were similar to those of the previous community structure differences (Figure 2). A heat map shows the hierarchical clustering of the top 40 genera under different treatments (Figure 3c,d). The microbial communities of the SC- and CF-treated samples were clustered separately, indicating that they exhibit distinct community characteristics. In terms of bacterial communities, the SC treatment was enriched with *Microvirga*, *Legionella*, *Ohtaekwangia*, *Polycyclovorans,* and *OM27_clade*, and the CF treatment was enriched with *Pseudomonas*, *Gemmatimonas*, *Paenibacillus,* and *Gaiella*. In terms of fungal communities, the SC treatment was enriched with *Myrmecridium*, *Fusicolla*, *Cystofilobasidium*, *Vishniacoizyima*, *Mucor*, and *Penicillium*, and the CF treatment was enriched with *llyoneciria*, *Mortierella*, *Leptosphaeria,* and *Torula*.

### 3.3. Correlation of Microbial Community with Functional Genes, Physicochemical Properties, and Enzyme Activities

**Figure 4:**
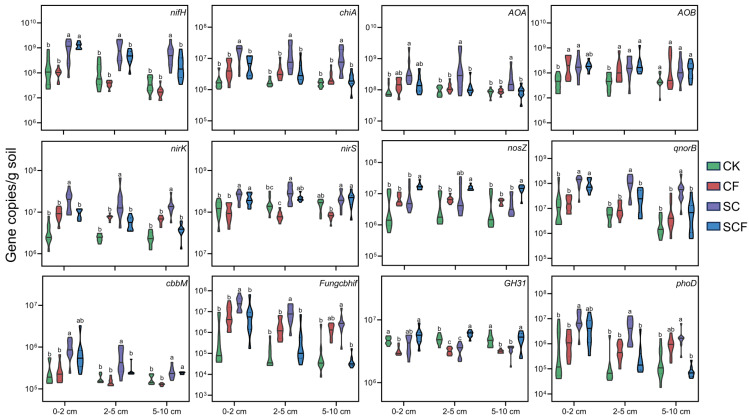
Changes in soil functional genes under different treatments. Different letters indicate sig-nificant differences (*p* < 0.05) among treatments. The eight nitrogen cycle genes were nitrogen fixation (*nifH*), organic nitrogen mineralization (*chiA*), ammonia oxidation (*AOA* and *AOB*), and denitrification (*nirK*, *nirS*, *nosZ* and *qnorB*). Three carbon cycle genes were carbon fixation (*cbbM*), cellulose degradation (*Fungcbhif*), and starch degradation (*GH31*). One phosphorus cycle gene was organic phosphorus mineralization (*phoD*).

The abundance of functional genes involved in soil carbon (C), nitrogen (N), and phosphorus (P) cycling increased under the different treatments (Figure 4 and Table S4). In terms of nitrogen cycling, the CF treatment only significantly increased the abundance of *AOB* genes, while the SC treatment enhanced the abundance of functional genes related to nitrogen fixation (*nifH*), organic nitrogen mineralization (*chiA*), nitrification (*AOA* and *AOB*), and denitrification (*nirK*, *nirS*, and *qnorB*) processes. Regarding carbon cycling and phosphorus cycling, the SC treatment significantly enhanced the abundance of functional genes related to carbon fixation (*cbbM*), cellulose degradation (*Fungcbhif*), and organic phosphorus mineralization (*phoD*). These results indicated that the SC treatment enhanced more microbial functional attributes.

**Figure 5:**
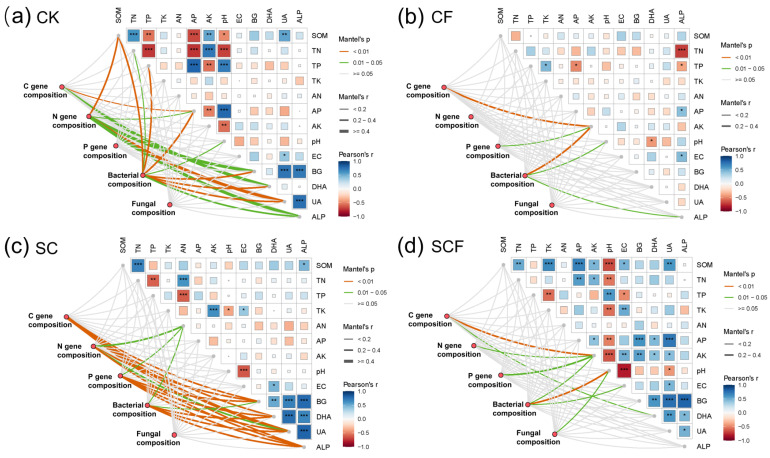
Mantel analyses of C, N, and P cycle genes and bacterial and fungal community composition (based on Bray–Curtis distance) with each edaphic factor under different treatments. *p* values were indicated by asterisks: * *p* < 0.05, ** *p* < 0.01 and *** *p* < 0.001. The width of the line corresponds to the Mantel r-value, and the color of the line indicates statistical significance. Heat map colors indicate Pearson correlations between environmental factors.

Based on the soil physicochemical properties and enzyme activities (Table S5), the Mantel test was used to further assess their correlation with microbial communities and functional gene composition (Figure 5). Following the CF treatment, AK was substantially correlated with C and P functional genes and bacterial community composition, while pH was significantly correlated with the bacterial community (Figure 5a,b). Meanwhile, pH was significantly correlated with both bacterial and fungal communities under the SCF treatment (Figure 5d). After the SC treatment, AN was significantly correlated with N and P functional genes and bacterial community composition (Figure 5c). All enzyme activity were shown to be substantially linked with the functional genes C, N, and P, as well as bacterial community composition. Furthermore, there was a significant positive association between enzyme activity. These results showed a strong association between biotic factors and microbial functional traits under the SC treatment. The VPA analyses further quantified the contribution of the bacterial and fungal communities to the functional gene variation in C, N, and P (Figure S5). The bacteria explained more of the functional variance than the fungi in all groups. Under the SC treatment, the bacteria could explain the highest amount of variance for C (34.3%), N (34.3%), and P (37.5%). This suggested that the bacterial community under the SC treatment was an important influencing factor.

### 3.4. Potential Microorganisms Driving Ecological Multifunctionality

**Figure 6:**
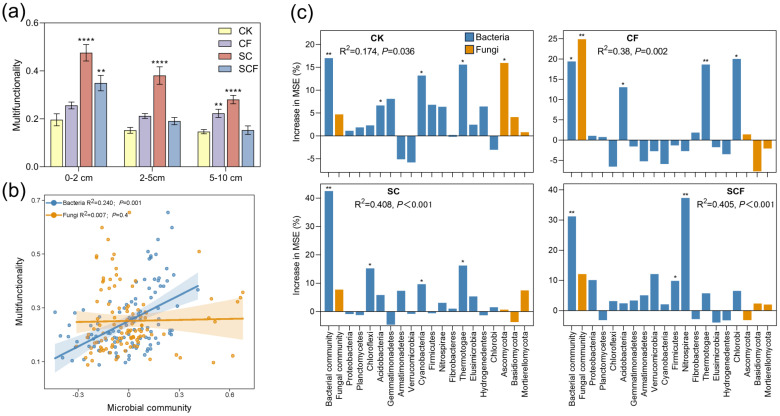
Bacterial and fungal communities in relation to soil multifunctionality among different treatments. *p* values were indicated by asterisks: * *p* < 0.05, ** *p* < 0.01 and **** *p* < 0.0001. (**a**) Differences in soil multifunctionality among treatments and depths. Error bars indicate standard deviation. Significance of difference between fertilizer group and control group was calculated. (**b**) Linear regression showed the relationship between microbial communities and soil multifunctionality. The first axis of NMDS represents microbial communities. (**c**) RF demonstrated the importance of bacterial community, fungal community, and dominant phylum (>1% of total community) as drivers for soil multifunctionality under different treatments. The percentage of mean square error (MSE%) indicates the importance of the driver.

To elucidate the impact of microbial composition on ecological functionality, we transformed the measured soil environmental factors and functional gene data into ecological multifunctionality. Simultaneously, we conducted a linear regression analysis for the microbial community (Figure 6). The SC treatment significantly enhanced soil ecological multifunctionality compared to that of the control (Figure 6a). In addition, its bacterial community (R^2^ = 0.656) fitted linearly with multifunctionality better than the fungal community (R^2^ = 0.012) (Figure S6). In contrast, the CF treatment enhanced ecological multifunctionality, but both microbial communities were poorly linearly fitted (Figure 6b). The RF treatment was used to investigate the primary microbial taxa that predict soil multifunctionality (Figure 6c). The contribution of the bacterial communities under the SC and SCF treatments is higher than that of the fungal communities, while the effect of the CF treatment is the opposite. Chloroflexi, Cyanobacteria, and Thermotogae were significant variables predicting multifunctionality in the SC treatment. Firmicutes and Nitrospirae were significant variables in the SCF treatment. Acidobacteria, Thermotogae, and Chlorobi were significant predictive variables for the CF treatment. The results of the RF analysis revealed seven significant bacterial phyla and one significant fungal phylum. Furthermore, we investigated their contributions at the genus level of classification (Figure S7). Interestingly, we found that important microbial genera of the SC and SCF treatments were also predictive variables of multiple ecological cycle functions (Figure S8). For example, *Herpetosiphon* in the SC treatment and *Nitrospira* in the SCF treatment were important predictive variables for C, N, and P cycle functions, and *Fusicolla* in the SC treatment was an important predictive variable for N and P cycles. However the important microbial genera of the CF treatment were predictive variables for only a single ecological cycle function. For example, *Sarocladium*, *Fusariella,* and *Blumeria* of the CF treatment were predictive variables for C, N, and P, respectively. The above results indicate that potentially important microbial taxa differ under different treatments and that they perform different ecological functions. Specifically, the SynCom significantly improved the soil’s carbon sequestration capacity, nitrogen conversion efficiency, and phosphorus effectiveness, functions that are critical for maintaining soil fertility and promoting plant growth. Furthermore, the SynCom enhanced the variety of soil microbial communities. Diverse microbial communities provide a wider range of ecological functions and enhance the ability of soils to withstand environmental stresses. These functions help increase soil productivity and support plant growth, which in turn increase vegetation cover and reduce the amount of bare surface area. Therefore, our study further highlights the potential value of using biotechnology such as SynComs in soil ecological restoration and desertification control.

## 4. Discussion

### 4.1. Fertilization Measures Have Altered Microbial Diversity and Composition

Soil microorganisms are important for maintaining soil ecological balance and promoting plant growth. The level of microbial alpha-diversity increased under the different fertilizer applications in desertified areas (Figure 2a,e). The difference was that the chemical fertilizer (CF) only enhanced the fungal alpha-diversity, whereas the SynCom (SC) and combined fertilization (SCF) treatments significantly enhanced the bacterial and fungal alpha-diversity. External additives selectively impact microbial groups [29]. The SynCom affected the soil fertility by directly increasing the population of beneficial microorganisms, so the alpha-diversity changed as expected. Whereas chemical fertilizers enhance soil nutrients through nutrient supplementation, bacterial and fungal growth preferences and adaptations influence changes in microbial diversity. Inadequate nutrient accumulation may be responsible for the lack of significant changes in microbial alpha-diversity. The NMDS plot demonstrates that the fertilizing measures had a greater impact on the microbial population than the soil depth (Figure 2b,f). Meanwhile, the bacterial community was more susceptible to changing conditions than the fungal community. This reflects the fact that bacteria respond faster to changes in external factors, while fungi respond more slowly. This phenomenon has also been demonstrated in many ecosystem studies [30], which may be due to the faster growth rate of bacteria.

A dissimilarity analysis showed that the SC treatment had a greater impact on the bacterial communities, while the CF treatment affected the fungal communities more significantly (Figure 2c,g). Interestingly, most of the bacterial genera enriched by the SC treatment are eutrophic bacteria. For example, *Microvirga, Legionella, Polycyclovorans, and OM27_clade* belong to the phylum Proteobacteria, and *Ohtaekwangia* belongs to the phylum Bacteroidetes (Figure 3c). Eutrophic bacteria can utilize abundant carbon sources for rapid growth and can be used in vegetation restoration [31]. However, this was not the case with the CF treatment, so the results suggest that the SC treatment altered the bacterial community in favor of the growth of eutrophic bacteria. Fungal communities are sensitive to CF [32,33], and our results are in agreement with this. The CF treatment increased the relative abundance of Mortierellomycota as a dominant phylum. Mortierellomycota often survive in nutrient-rich soils as saprophytes and have been strongly correlated with soil pH, nitrogen, and phosphorus nutrients in previous studies [34]. Combined fertilizer applications can avoid the problems of nutrient imbalance and poor structure associated with single applications [35]. In this study, the SCF treatment had the highest bacterial and fungal community differences compared to the CK treatment. This result suggests that the SCF treatment integrates the effects of two single fertilizer applications, proving highly beneficial in terms of fertilizer reduction and cost reduction.

### 4.2. SynCom Enhanced the Link between Microorganisms and Soil Biological Properties

The composition of species can reflect the abundance changes in microbial communities. However, it should be noted that microorganisms within the same taxonomic group may not necessarily possess identical ecological functions. For example, different strains of the genus *Pseudomonas* exhibit significant differences in material degradation and plant growth promotion [36]. Therefore, we determined some C, N, and P cycle-related functional genes to understand the functional characteristics of microbial taxa (Figure 4 and Table S4). The SC treatment significantly enhanced nitrogen fixation, nitrogen mineralization, nitrification, and denitrification processes in the N cycle. This suggests that soil microbial action promotes the N cycle. Microorganisms have also been demonstrated to play a vital role in controlling the nitrogen cycle in previous grassland restoration studies, in which a general increase in N inputs boosted vegetation restoration [37]. The *AOA* and *AOB* genes characterized the ammonia oxidation process of nitrification, and different types of ammonia-oxidizing microbial communities differed in terms of their sensitivity to the environment. The CF treatment only significantly increased *AOB* gene abundance, suggesting that it promoted nitrified nitrogen acquisition by enhancing ammonia-oxidizing bacteria. This result is consistent with that in the study of Allegrini et al. [38], which showed *AOB* genes are the main responders to the action of inorganic fertilizers. Here, we have found that the SC treatment has a universal effect on enhancing soil functional characteristics, which may be attributed to the reshaping of the microbial community, enabling multifunctional collaboration. C fixation and C degradation by microorganisms are important in maintaining soil C balance. In the present study, the SC treatment significantly enhanced cellulose degradation (*Fungcbhif* gene) and reduced starch degradation (*GH31* gene). This suggests that the functional characteristics of microorganisms are influenced by nutrient limitations. Most of the C sources in barren environments are recalcitrant, which requires oligotrophic microorganisms for degradation to release nutrients [39,40]. Therefore, in the initial stage of vegetation restoration, it is beneficial to enhance nutrient accumulation and plant growth by enhancing the utilization of complex organic matter. In summary, the SC treatment significantly enhanced various functional characteristics, promoting nutrient restoration and vegetation growth in desertified areas. Additionally, we also found that the SC treatment maintained the functional stability of the soil surface, whereas many functional characteristics of the SCF treatment were affected by the soil depth. For example, the *nifH*, *qnorB*, and *phoD* genes were enhanced only at 0–2 cm by the SCF treatment and weakened by depth changes. These results suggest that the combined fertilization treatment may have weakened the impact of synthetic flora due to nutrient conflicts.

Mantel visualizations were used to explore the links between microbial taxa and functional gene composition and soil factors (Figure 5). In this study, AK and pH were the main influences on microbial community and function under the CF treatment. They had the same effect under the SCF treatment. This may be due to soil acidification caused by the addition of chemical fertilizer. Some studies have shown that pH is correlated with soil aggregate stability and affects carbon fluxes and available nutrients in desert grasslands [41,42]. The heat maps show that pH was also significantly correlated with seven soil properties, such as SOM and TN (Figure 5d), emphasizing pH as a key factor influencing microbial communities. In contrast, AN was an important influencing factor under the SC treatment. The enhancement of functional genes for several nitrogen cycling processes in this study suggests that the microbial regulation of N turnover is closely linked. The above results suggest that the SC and CF treatments used different nutrient strategies to influence the microbial communities and their functional traits.

All enzyme activities significantly impacted microbial community composition and functional traits under the SC treatment. However, this was not found under the other treatments. This suggests that biotic factors are the main mode of action of microbial stimulation. Additionally, enzyme activity regulates nutrient cycling because most functional genes are involved in encoding the corresponding extracellular enzymes [43]. Numerous studies have demonstrated that beneficial microbial inoculation promotes enzyme activity in soil [44], and our study agrees with this view.

### 4.3. SynCom Increased the Contribution of Bacterial Communities to Soil Multifunctionality

Our VPA analyses (Figure S5) demonstrated how much the functional genes could be explained by the microbial community. In terms of C, N, and P cycling, the residuals of the CF treatment were similar to those of the CK treatment, while the SC treatment had the lowest residuals. The findings indicated that the SC treatment significantly enhanced the effect of the microbial community on multiple functional traits. This is consistent with our hypothesis that microbial communities play a greater role in soil functional improvement under SC treatments. Most studies have also confirmed that microbial taxa are significantly correlated with some ecological functions [45,46]. In this study, we suggest that the SC treatment may promote the effectiveness and synergy of microbial functions through the high adaptability of native microorganisms. Furthermore, we discovered that the bacterial community explained more than the fungal community, implying that the SC treatment may have enhanced the role of the bacterial community in restoring ecological services. In order to avoid the homogeneity of soil functions, we combined a variety of known soil indicators (enzyme activity, physicochemical properties, and functional gene abundance) to generate a multifunctionality index to explore the relationship between microbial communities and ecological multifunctionality in detail.

Different agricultural management practices can enhance soil multifunctionality. Our results were in agreement with this, in that all of the fertilization treatments increased the multifunctionality index (Figure 6a). The SC treatment significantly enhanced soil multifunctionality at different depths in the top soil layer (0–10 cm). However, the CF treatment significantly increased multifunctionality only at 5–10 cm. This indicates that the SC treatment can effectively enhance soil multifunctionality in desertified areas, contributing to ecological restoration. There is growing evidence that microorganisms play a key role in regulating ecosystem functions [47]. The positive relationship between biodiversity and soil multifunctionality has been widely recognized, with specific microbial taxa driving multiple soil functions [48,49]. However, the effect of microbial community changes on ecological multifunctionality under different fertilization conditions remains unclear. Liu et al. [8]’s research has demonstrated that litter crusts in desert areas enhance soil nutrients by affecting bacterial communities but not fungal communities. The present study also proved that bacterial communities are more closely related to multifunctional indicators in desertified areas. Bacterial communities were linearly correlated with multifunctionality, whereas the correlation was weaker for fungi (Figure 6b). The SC treatment enhanced the linear relationship between the bacterial communities and functionality. A random forest model also confirmed that the SC treatment increased the importance of the bacterial communities. Therefore, the results are consistent with our second hypothesis that the bacterial community under the SynCom treatment plays a major role in soil multifunctional restoration. Bacteria exhibit a wide range of traits and functions, and they grow rapidly [17]. Therefore, SynComs are proven to be an effective measure for ecological restoration by regulating bacterial communities.

### 4.4. Potentially Functional Microbial Taxa Play an Important Role in Ecological Multifunctionality

Microorganisms are key drivers of biogeochemical cycles. The random forest model demonstrated potential microbial predictors under the different treatments. Interestingly, key microbial genera under the SC and SCF treatments drove multiple elemental cycles, whereas key microbial genera under the CF treatments drove single elemental cycles. *Herpetosiphon* spp. are a class of filamentous slithering predatory bacteria capable of producing a wide range of secondary metabolites and hydrolytic enzymes [50]. The predator is able to selectively lyse its prey to release nutrients that promote the growth of other microorganisms. As a result, they play a significant role in reshaping microbial communities. Previous studies have reported that fertilization with added nutrients had less of an effect on microbial communities than predators [51]. This also explained why the SynCom did not introduce excessive nutrients, but was able to significantly change the structure of bacterial and fungal communities. *Fusicolla* spp. are important inter-root fungi and have an important role in biodegradation and plant promotion [52]. Zhu et al. [53] found a positive correlation between Fusicolla and soil nitrogen and phosphorus, which is consistent with our findings. These potentially key microorganisms can participate in multiple nutrient cycles, occupy a wide range of ecological niches, and enhance soil nutrient multifunctionality. These results suggest that SynCom highlights the importance of potentially functional taxa in ecological multifunctionality. Future studies should focus more on the physiological activities and metabolic characteristics of these multifunctional microbial taxa. This knowledge is crucial for us to apply microbial strategies for environmental remediation.

## 5. Conclusions

In conclusion, this study demonstrated that in desertified areas, the SynCom treatment was more effective for restoring soil multifunctionality compared to chemical fertilizers, with bacterial communities playing a major role. The SynCom induced significant changes in the bacterial communities, while the chemical fertilizers primarily affected fungal communities. While the chemical fertilizers increased the amount of soil nutrients, their association with microbial communities was weak. The SynCom significantly increased functional gene abundance, with biotic factors being the main drivers of microbial community and functional traits. The bacterial community under the SynCom was the primary driver of soil multifunctionality, with most potential microbial taxa being functionally diverse. The combined fertilization treatment shared microecological traits with the SynCom treatment and could be used for chemical fertilizer reduction in the future. Overall, our results highlight the positive impact of SynComs on microbial communities and ecological functions in desertified areas, providing insights into the use of microbial approaches to remediate soil environments.

## Data Availability

We have submitted all of our sequence data to the Beijing Institute of Genomics (BIG) database at https://ngdc.cncb.ac.cn/gsa/ accessed on 27 May 2024. The reference project number is PRJCA026503. We have ensured the public availability of these data so that other researchers can access and use them.

## Supplementary Materials

The following supporting information can be downloaded at: https://www.mdpi.com/article/10.3390/microorganisms12061117/s1, Figure S1: The design process of synthetic bacterial community; Table S1: Detail of PGPR candidates of SynCom; Figure S2: The rarefaction curves of bacterial and fungal communities; Table S2: The relative abundance of bacterial phyla (top 10) in the four treatment groups and three different soil layers; Table S3: The relative abundance of fungal phyla (top 10) in the four treatment groups and three different soil layers; Figure S3: Bacterial differential ASVs under different treatments; Figure S4: Fungal differential ASVs under different treatments; Table S4: Primer information and reaction procedures for soil functional genes; Table S5: Soil physicochemical properties and enzyme activities under different treatments; Figure S5: The interpretation of function gene composition by bacterial and fungal communities under different treatments; Figure S6: Linear regression of bacterial and fungal community to soil multifunctionality; Figure S7: RF shows the importance of bacterial and fungal genera for soil multifunctionality under different treatment groups; Figure S8: Calculation of the importance of bacterial and fungal genus distributions for the multifunctionality of the C, N, and P cycles, respectively, based on RF model.
