# Supplementary material for: Synthetic Microbial Community Promotes Bacterial Communities Leading to Soil Multifunctionality in Desertified Land
Source: Microorganisms. 2024 May 30;12(6):1117. doi: 10.3390/microorganisms12061117 (PMC11205429; doi:10.3390/microorganisms12061117)
Supplement: Supplementary file 1 [file microorganisms-12-01117-s001.zip › microorganisms-3019058-supplementary.pdf]

Article

Synthetic microbial community promotes bacterial communities driving soil multifunctionality in desertified land

Xinwei Hao [1], Yazhou Gu [2,*], Hongzhi Zhang [2], Xiao Wang [1], Xiaozhen Liu [3], Chunlei Chen [1], Congcong Wang [1], Xiaoqing Zhang [3], Xingyu Liu [4] and Xihui Shen [1,*]

[1] State Key Laboratory for Crop Stress Resistance and High-Efficiency Production, Shaanxi Key Laboratory of Agricultural and Environmental Microbiology, College of Life Sciences, Northwest A&F University, Yangling, Shaanxi 712100, P. R. China; xinweihao1995@163.com (X.H.); wangxiaoyx@nwafu.edu.cn (X.W.); 15612250872@163.com (C.C.); wangcc@nwafu.edu.cn (C.W.)

[2] Qingyang Longfeng Sponge City Construction Management and Operation Co., Ltd., Qingyang 745000, China; 15688943689@163.com (H.Z.)

[3] Institute of Grassland Research, Chinese Academy of Agricultural Sciences, Hohhot 010013, China; xiaozhenliu88@163.com (X.L.); zhangxiaoqing@caas.cn (X.Z.)

[4] State Key Laboratory of Geological Processes and Mineral Resources, Institute of Earth Sciences, China University of Geosciences, Beijing 100083, China; wellwoodliu@163.com (X.L.)

* Correspondence: gyz0916@sina.com (Y.G.); xihuishen@nwsuaf.edu.cn (X.S.)

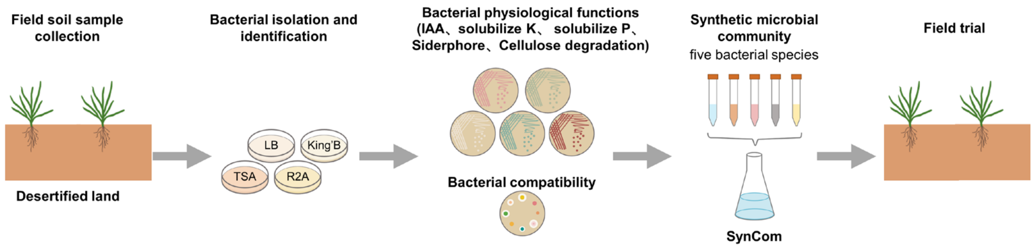

FIGURE S1 The design process of synthetic bacterial community. Plant inter-root soil from desertified soil was collected for strain isolation. Afterwards, the combination of the flora was optimized by functional identification of the strains and compatibility experiments. In this study, a synthetic colony consisting of five plant growth-promoting strains was constructed. Finally, the synthetic flora was applied to the field experiment to evaluate the effect.

TABLE S1 Detail of PGPR candidates of SynCom.

SynCom PGPR candidates	IAA	Phosphorus solubilisation	Potassium solubilisation	Siderophore production	Cellulose degradation	Compatibility
Arthrobacter sp.	+	+	+		+	+
Bacillus sp.		+		+	+	+
Acinetobacter sp.		+	+		+	+
Pseudomonas sp.	+	+		+		+
Mixta sp.		+	+	+		+

PGPR, plant growth-promoting rhizobacteria; IAA, indole acetic acid production.

(a) (b)

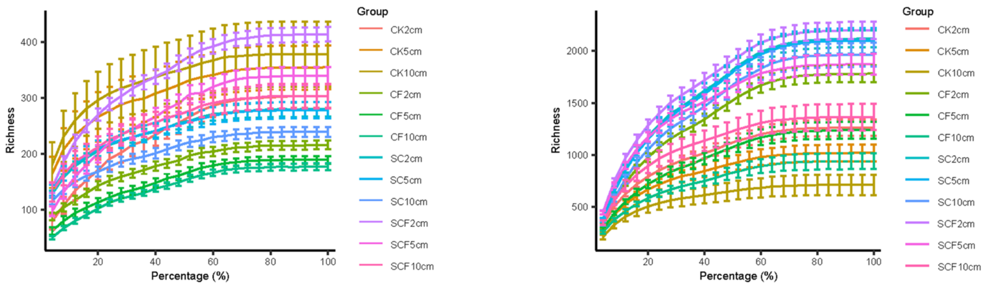

FIGURE S2 The rarefaction curves of bacterial (a) and fungal (b) communities. Error bars indicate standard deviation. The twelve subgroups included four treatments and three soil layers. CK, no fertilizer; CF, chemical fertilizer; SC, SynCom; SCF, combined SynCom-chemical fertilizer.

TABLE S2 The relative abundance of bacterial phyla (top 10) in the four treatment groups and three different soil layers.

Phyla	CK			CF			SC			SCF		
	0-2cm	2-5cm	5-10cm	0-2cm	2-5cm	5-10cm	0-2cm	2-5cm	5-10cm	0-2cm	2-5cm	5-10cm
Proteobacteria	35.66	38.84	39.97	31.34	35.45	38.95	34.51	38.36	41.75	29.25	33.96	34.67
Actinobacteria	32.92	35.82	40.09	37.12	38.87	34.92	34.36	34.29	33.65	36.11	34.05	34.69
Planctomycetes*	10.38	5.51	3.19	9.20	5.36	3.80	7.15	6.93	5.23	9.51	8.50	7.26
Bacteroidetes	6.55	6.27	5.64	7.29	6.46	6.65	7.29	5.99	7.05	8.75	6.96	6.15
Acidobacteria*	4.38	4.39	3.94	6.76	5.27	4.95	6.14	5.43	4.23	5.66	6.59	6.79
Chloroflexi*	2.74	2.01	1.68	1.82	1.81	3.14	3.50	3.11	2.59	3.76	2.99	2.39
Gemmatimonadetes*	2.29	1.58	1.60	1.94	1.90	2.02	1.73	1.55	1.32	2.03	1.84	1.88
Cyanobacteria	1.90	2.00	0.70	0.85	0.78	1.03	1.08	0.72	0.80	1.73	1.01	1.16
Verrucomicrobia*	0.60	0.54	0.43	1.62	1.69	0.62	1.69	1.23	1.32	1.06	1.48	1.44
Other	2.58	3.04	2.76	2.05	2.41	3.91	2.56	2.39	2.07	2.15	2.62	3.58

The asterisk (*) indicates the presence of significant ($p < 0.05$) differences among fertilizer treatments. CK, no fertilizer; CF, chemical fertilizer; SC, SynCom; SCF, combined SynCom-chemical fertilizer.

TABLE S3 The relative abundance of fungal phyla (top 10) in the four treatment groups and three different soil layers.

Phyla	CK			CF			SC			SCF		
	0-2cm	2-5cm	5-10cm	0-2cm	2-5cm	5-10cm	0-2cm	2-5cm	5-10cm	0-2cm	2-5cm	5-10cm
Ascomycota*	84.83	57.41	59.23	21.04	33.32	42.62	35.84	21.95	28.09	44.51	43.84	43.17
Unassigned*	11.74	15.23	24.08	34.47	19.62	9.18	53.43	71.44	59.86	41.80	47.44	23.96
Basidiomycota	0.67	23.54	13.20	2.16	1.96	1.45	6.28	3.61	3.13	4.89	3.75	13.83
Mortierellomycota*	2.54	1.92	2.94	42.33	45.08	46.74	4.20	2.89	8.76	8.25	4.86	18.46
Chytridiomycota	0.21	1.82	0.53	0.00	0.00	0.00	0.01	0.00	0.00	0.55	0.10	0.47
Rozellomycota	0.00	0.05	0.01	0.00	0.00	0.00	0.00	0.00	0.00	0.00	0.00	0.01
Mucoromycota	0.00	0.00	0.01	0.00	0.02	0.00	0.01	0.07	0.07	0.01	0.00	0.08
Glomeromycota*	0.00	0.00	0.00	0.00	0.00	0.00	0.23	0.05	0.09	0.00	0.00	0.00
Blastocladiomycota	0.00	0.02	0.01	0.00	0.00	0.00	0.00	0.00	0.00	0.00	0.00	0.00
Zoopagomycota	0.00	0.00	0.00	0.00	0.00	0.00	0.00	0.00	0.00	0.00	0.00	0.00

The asterisk (*) indicates the presence of significant ($p < 0.05$) differences among fertilizer treatments. CK, no fertilizer; CF, chemical fertilizer; SC, SynCom; SCF, combined SynCom-chemical fertilizer.

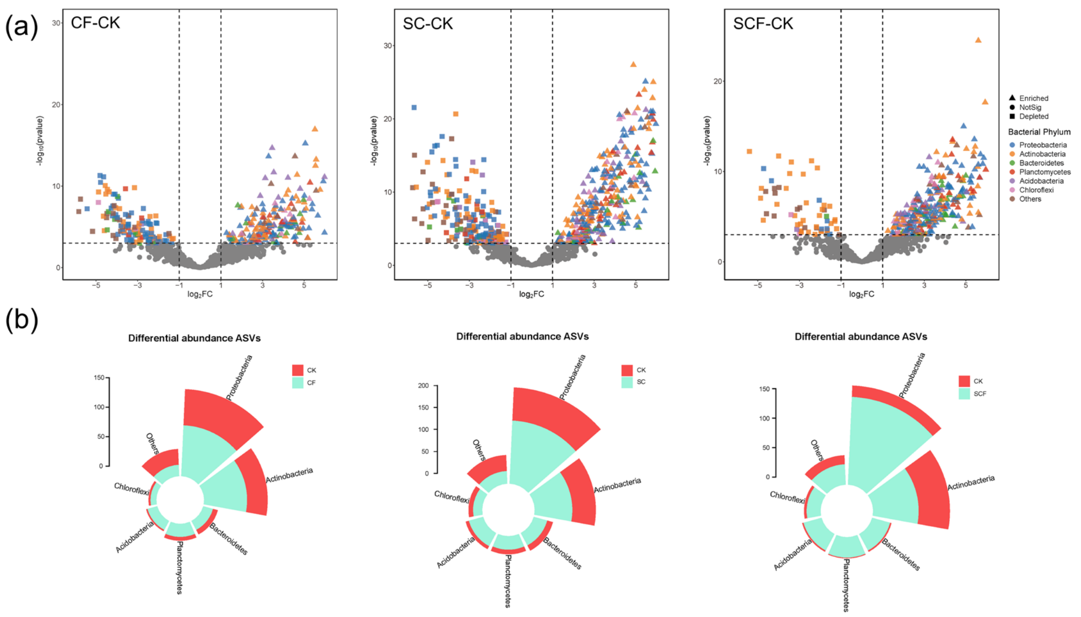

FIGURE S3 The volcano plots (a) indicate bacterial differential ASVs under different treatments. Dot colors indicate phylum level classification. Dot traits indicate enrichment or deletion. The stacked bar plots (b) indicate the percentage of differential ASVs at the phylum level under different treatments. CK, no fertilizer; CF, chemical fertilizer; SC, SynCom; SCF, combined SynCom-chemical fertilizer.

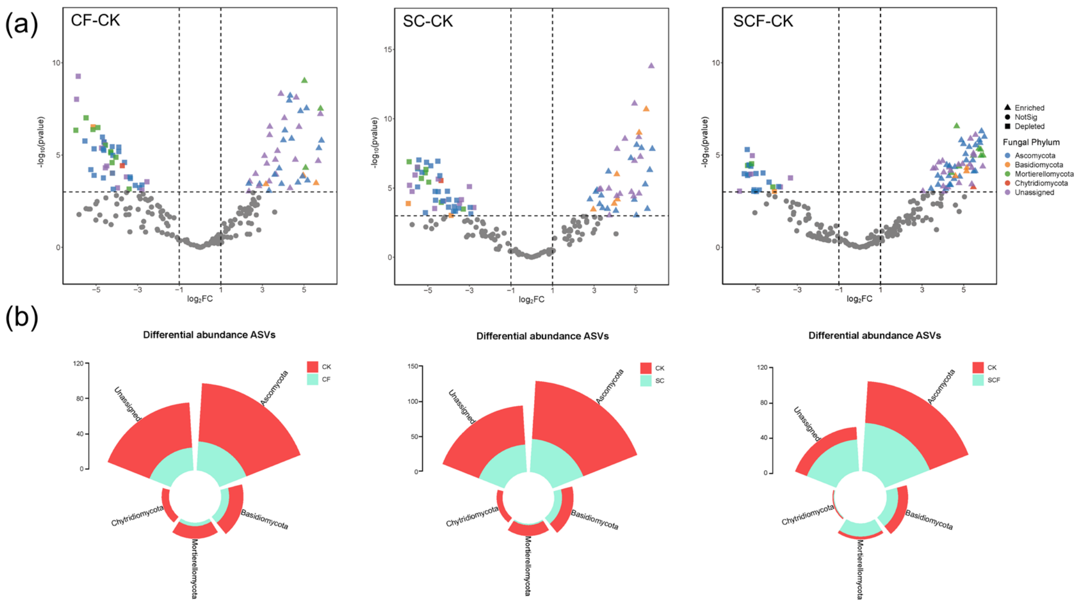

FIGURE S4 The volcano plots (a) indicate fungal differential ASVs under different treatments. Dot colours indicate phylum level classification. Dot traits indicate enrichment or deletion. The stacked bar plots (b) indicate the percentage of differential ASVs at the phylum level under different treatments. CK, no fertilizer; CF, chemical fertilizer; SC, SynCom; SCF, combined SynCom-chemical fertilizer.

TABLE S4 Primer information and reaction procedures for soil functional genes

Target gene	Primers name	Primer sequence (5'-3')	Thermal profile
**N cycle function gene**			
AOA	CamoA-19F	ATGGTCTGGYTWAGACG	95°C 5 min; 95°C 30 s, 50°C 60 s, 72°C 60 s; 35 cycles
	CamoA-616R	GCCATCCABCKRTANGTCCA	
AOB	*amoA*-1F	GGGGTTTCTACTGGTGGT	94°C 5 min; 94°C 30 s, 55°C 45 s, 72°C 60 s; 40 cycles
	amoA-2R	CCCCTCKGSAAAGCCTTCTTC	
nirK	*nirK*583F	TCATGGTGCTGCCGCGKGACGG	95°C 10 min; 95°C 15 s, 64°C 45 s, 72°C 30 s; 40 cycles
	*nirK*909R	GAACTTGCCGGTKGCCCAGAC	
nirS	*nirS*cd3aF	GTSAACGTSAAGGARACSGG	95°C 10 min; 95°C 15 s, 64°C 45 s, 72°C 30 s; 40 cycles
	*nirS*R3cd	GASTTCGGRTGSGTCTTGA	
nosZ	*nosZ* 1527F	CGCTGTTCHTCGACAGYCA	95°C 10 min; 95°C 15 s, 58°C 50 s, 72°C 30 s; 40 cycles
	nosZ 1773R	ATRTCGATCARCTGBTCGTT	
nifH	*nifH* F	AAAGGYGGWATCGGYAARTCCACCACTT	95°C 10 min; 95°C 15 s, 54°C 50 s, 72°C 30 s; 40 cycles
	nifH R	GTTSGCSGCRTACATSGCCATCAT	
qnorB	*qnorB*2F	GGNCAYCARGGNTAYGA	95°C 5min; 95°C 20s, 57°C 30s, 72°C 30s; 46 cycles
	*qCDRnorB*5R	ACCCANAGRTGNACNACCCACCA	
**C cycle function gene**			
chiA	*GA1*F	CGTCGACATCGACTGGGARTDBCC	94°C 10min; 94°C 30s ;55°C 30s; 72°C 30s; 35 cycles
	*GA1*R	ACGCCGGTCCAGCCNCKNCCRTA	
cbbM	*cbbM-F*	GGCACCATCATCAAGCCCAAG	94°C 5 min; 94°C 30 s, 55°C 45 s, 72°C 60 s; 35 cycles
	cbbM-R	TCTTGCCGTAGCCCATGGTGC	
Fungcblf	*fungcblF*	ACCAAYTGCTAYACIRGYAA	95°C 10 min; 95°C 30 s, 48°C 45 s, 72°C 90 s; 40 cycles
	fungcblR	GCYTCCCAIATRTCCATC	
GH31	*350F*	CAYCARTGYMGITGGGGNTA	95°C 10 min; 95°C 45 s, 50°C 45 s, 72°C 100 s; 40 cycles
	660R	TTRTCICCNCCCCARTGNCC	
**P cycle function gene**			
phoD	*ALPS-F730*	CAGTGGGACGACCACGAGGT	95 °C 30s, 60°C 5s, 72°C 34s; 40 cycles
	ALPS-1101	GAGGCCGATCGGCATGTCG	

TABLE S5 Soil physicochemical properties and enzyme activities under different treatments

Treatment	Sand layer (cm)	Organic M (g/kg)	pH (1:2.5 H_2O)	EC (ds/m)	Total N (g/kg)	Total P (g/kg)	Total K (g/kg)	Availiable N (mg/kg)
CK	0-2	4.24±0.18 Ba	8.66±0.02 Ba	0.15±0.004 Ba	0.30±0.01 Ba	0.30±0.02 Ba	16.27±0.27 Aa	5.37±0.43 Aa
	2-5	4.15±0.16 Ba	8.67±0.02 Ba	0.13±0.002 Bb	0.31±0.01 Ba	0.31±0.03 Ba	15.92±0.57 Aa	4.69±0.25 Aa
	5-10	4.21±0.13 Ca	8.67±0.02 Aa	0.13±0.002 Bb	0.30±0.01 Ba	0.31±0.03 Ba	16.67±0.33 Aa	5.19±0.40 Ba
CF	0-2	5.65±0.12 Aa	8.69±0.01 ABa	0.15±0.003 Ba	0.33±0.004 ABa	0.40±0.01 Ab	15.36±0.30 Ab	7.13±0.90 Aa
	2-5	5.40±0.22 Aa	8.70±0.02 ABa	0.14±0.003 Bab	0.33±0.01 ABa	0.43±0.01 Aab	16.19±0.10 Aa	6.41±0.29 Aa
	5-10	5.58±0.15 Aa	8.68±0.02 Aa	0.14±0.002 ABb	0.33±0.009 Ba	0.44±0.006 Aa	15.85±0.19 ABab	7.25±0.72 ABa
SC	0-2	6.02±0.25 Aa	8.75±0.02 Aa	0.23±0.04 Aa	0.40±0.02 Aa	0.43±0.01 Aa	16.05±0.18 Aa	6.92±0.57 Aa
	2-5	5.86±0.22 Aa	8.74±0.01 Aa	0.21±0.01 Aa	0.40±0.02 Aa	0.44±0.02 Aa	15.80±0.25 Aa	7.50±0.58 Aa
	5-10	5.51±0.23 Aa	8.73±0.02 Aa	0.21±0.02 Aa	0.38±0.02 Aa	0.44±0.01 Aa	15.79±0.29 ABa	8.21±0.52 Aa
SCF	0-2	5.32±0.38 ABa	8.61±0.03 Ba	0.17±0.01 ABa	0.37±0.03 ABa	0.31±0.01 Ba	14.59±0.81 Aa	5.75±0.55 Aa
	2-5	4.93±0.26 ABa	8.64±0.03 Ba	0.15±0.006 ABa	0.35±0.03 ABa	0.31±0.01 Ba	15.00±0.65 Aa	5.78±0.38 Aa
	5-10	4.92±0.27 Ba	8.67±0.04 Aa	0.15±0.007 ABa	0.34±0.02 Ba	0.31±0.01 Ba	14.80±0.41 Ba	5.82±0.41 Ba

Treatment	Sand layer (cm)	Availiable P (mg/kg)	Availiable K (mg/kg)	BG (μg PNP g^{-1} h^{-1})	DHA (μg TPF g^{-1} h^{-1})	UA (μg NH_3-N g^{-1} d^{-1})	ALP (μg PNP g^{-1} h^{-1})
CK	0-2	9.11±1.42 Ba	154.00±8.25 Aa	0.08±0.04 Ba	0.19±0.08 Ba	0.49±0.12 Ba	1.71±0.22 Ba
	2-5	8.46±1.42 Ba	149.00±7.88 Aa	0.03±0.02 Ba	0.34±0.14 Ba	0.24±0.03 Bb	1.48±0.08 ABa
	5-10	8.76±1.53 Ba	143.56±4.60 Aa	0.02±0.007 Ba	0.26±0.17 ABa	0.25±0.03 Cb	1.47±0.13 ABa
CF	0-2	20.07±2.41 Aa	136.89±4.63 Aa	0.05±0.01 Ba	0.07±0.03 Ba	0.38±0.03 Ba	1.37±0.06 Ba
	2-5	19.64±2.31 Aa	118.22±4.05 Ab	0.03±0.005 Ba	0.005±0.005 Ba	0.36±0.04 Ba	1.36±0.07 Ba
	5-10	17.60±0.50 Aa	121.33±4.78 Bb	0.06±0.02 ABa	0.02±0.01 Bab	0.41±0.06 Ba	1.26±0.07 ABa
SC	0-2	17.90±1.50 Aa	144.67±10.35 Aa	0.36±0.05 Aa	1.31±0.22 Aa	1.48±0.19 Aa	2.19±0.18 Aa
	2-5	17.38±1.52 Aa	130.11±4.25 Aa	0.38±0.10 Aab	0.85±0.22 Aa	1.15±0.15 Aa	1.83±0.21 Aab
	5-10	21.73±2.49 Aa	122.33±7.30 Ba	0.16±0.04 Ab	0.15±0.04 Ab	0.62±0.06 Ab	1.50±0.10 Ab
SCF	0-2	13.45±1.08 ABa	152.78±9.35 Aa	0.30±0.06 Aa	0.69±0.19 ABa	0.85±0.09 ABa	1.91±0.18 ABa
	2-5	11.72±0.49 ABa	120.00±10.75 Ab	0.04±0.01 Ab	0.10±0.04 ABb	0.49±0.05 ABb	1.44±0.07 ABb
	5-10	10.87±0.41 Ba	117.89±6.62 Bb	0.01±0.01 Bb	0.06±0.04 ABb	0.36±0.04 BCb	1.20±0.04 Cb

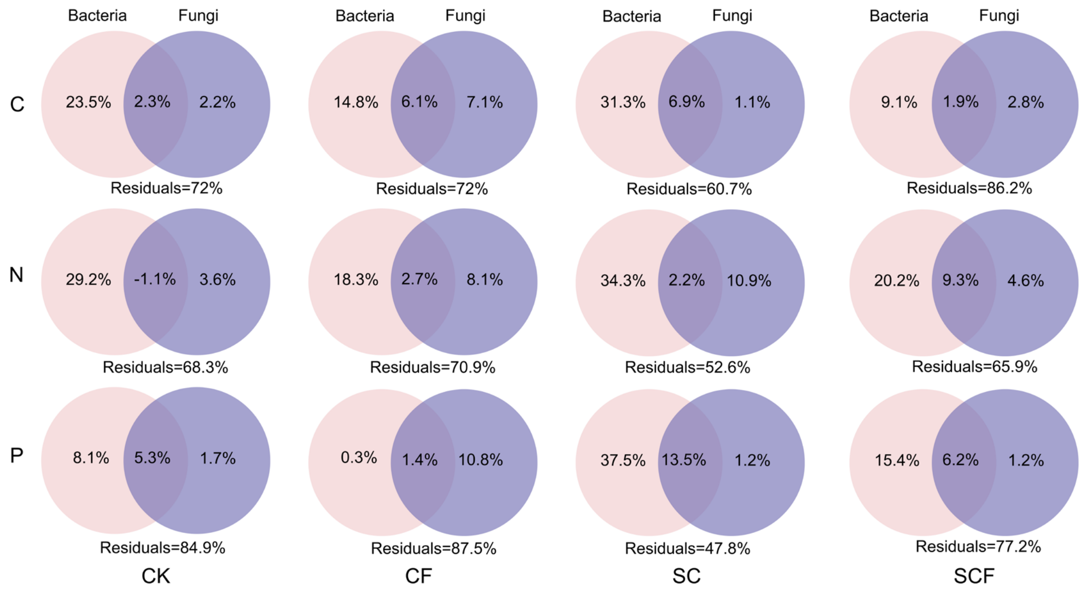

FIGURE S5 Variation partitioning analysis (VPA) represents the interpretation of C, N and P cycle function gene composition by bacterial and fungal communities under different treatments. Residuals represent unexplained proportions. C, carbon cycle. N, nitrogen cycle. P, phosphorus cycle. CK, no fertilizer; CF, chemical fertilizer; SC, SynCom; SCF, combined SynCom-chemical fertilizer.

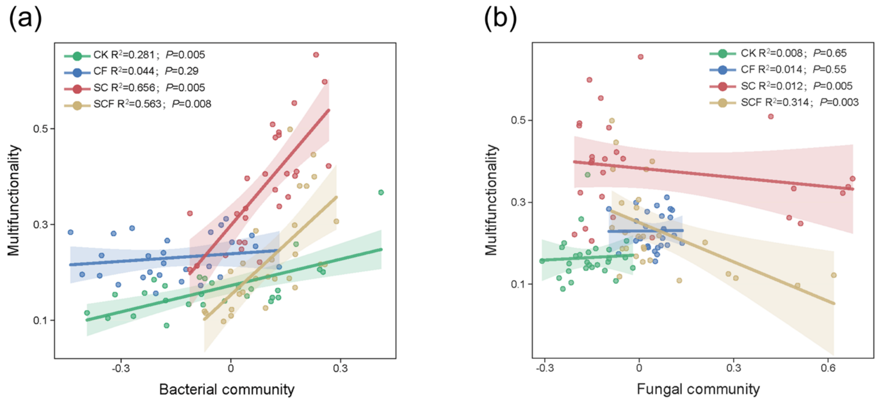

FIGURE S6 Linear regression of bacterial (a) and fungal (b) community to soil multifunctionality. The first axis of NMDS represents microbial community structure. R^2 denotes the linear regression coefficient of determination, and a larger value indicates a better fit. The p-value represents the significance of the linear fit, with less than 0.05 indicating significance. CK, no fertilizer; CF, chemical fertilizer; SC, SynCom; SCF, combined SynCom-chemical fertilizer.

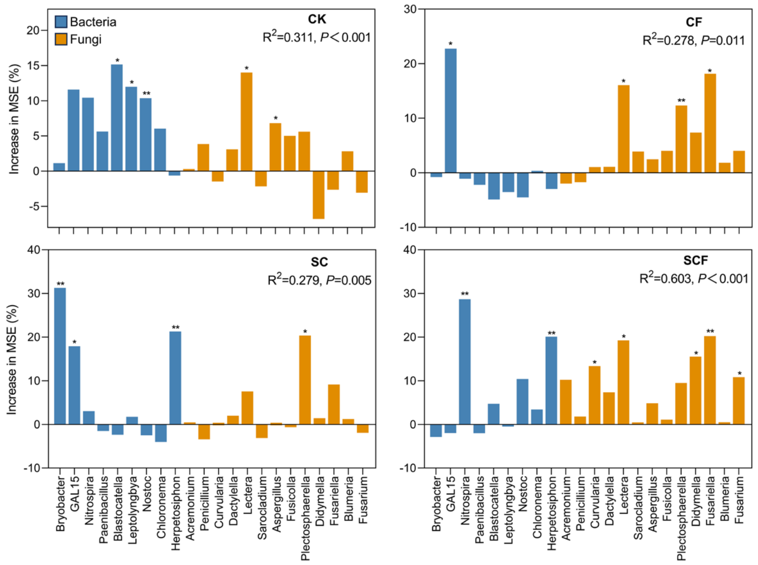

FIGURE S7 The Random Forest (RF) shows the importance of bacterial and fungal genera for soil multifunctionality under different treatment groups. These genera are all from phyla with significant driving roles. Blue columns indicate bacterial genera and yellow columns indicate fungal genera. MSE is the mean square error. MSE% values represent the significance of these predictors. The higher the MSE% value, the more important the predictor. The asterisks on the column represent significance (*p < 0.05, **p < 0.01, and ***p < 0.001). *P* represents the model significance and R^2 indicates the explanatory rate of the model. CK, no fertilizer; CF, chemical fertilizer; SC, SynCom; SCF, combined SynCom-chemical fertilizer.

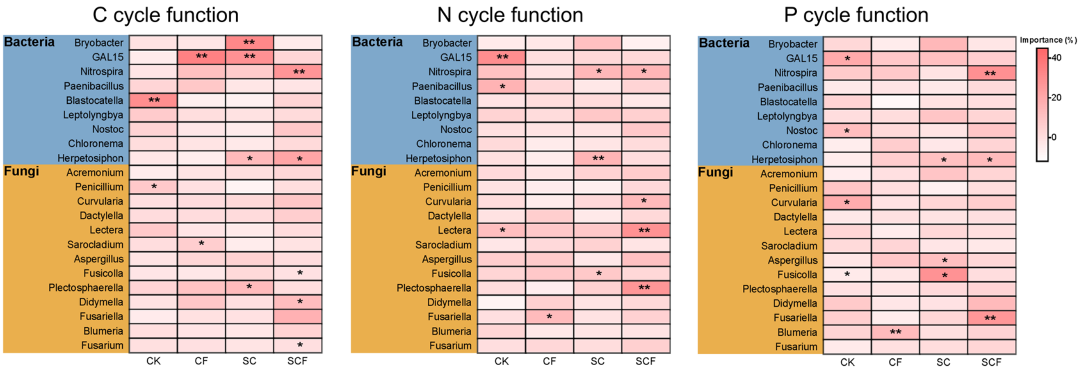

FIGURE S8 Calculation of the importance of bacterial and fungal genus distributions for the multifunctionality of the C, N, and P cycles, respectively, based on random forest (RF) model. The darker color of the small square in the heat map indicates the higher importance of the genus. The asterisks on the column represent significance (*p < 0.05, **p < 0.01, and ***p < 0.001). All RF models were significant under different treatments. C, carbon cycle. N, nitrogen cycle. P, phosphorus cycle. CK, no fertilizer; CF, chemical fertilizer; SC, SynCom; SCF, combined SynCom-chemical fertilizer.
